# Effects of Fumonisin B and Hydrolyzed Fumonisin B on Growth and Intestinal Microbiota in Broilers

**DOI:** 10.3390/toxins14030163

**Published:** 2022-02-23

**Authors:** Song Yu, Bingxuan Jia, Huikang Lin, Shuo Zhang, Dianzhen Yu, Na Liu, Aibo Wu

**Affiliations:** 1 SIBS-UGENT-SJTU Joint Laboratory of Mycotoxin Research, CAS Key Laboratory of Nutrition, Metabolism and Food Safety, Shanghai Institute of Nutrition and Health, University of Chinese Academy of Sciences, Shanghai 200031, China; jiabingxuan2017@sibs.ac.cn (B.J.); linhuikang2020@sibs.ac.cn (H.L.); dzyu@sibs.ac.cn (D.Y.); liuna@sibs.ac.cn (N.L.); 2 Division of Chemical Toxicity and Safety Assessment, Shanghai Municipal Center for Disease Control and Prevention, Shanghai 200336, China; syu@sibs.ac.cn; 3 NHC Key Laboratory of Food Safety Risk Assessment, China National Center for Food Safety Risk Assessment, Beijing 100021, China; zhangshuo6789@sina.com

**Keywords:** fumonisin B, hydrolyzed fumonisin B, growth performance, intestinal microbiota, broiler

## Abstract

Fumonisins are mainly produced by *Fusarium verticillioides* and *proliferatum*, which causes a variety of toxicities in humans and animals, including fumonisin Bs (FBs) as the main form. After they are metabolized by plants or microorganisms, modified fumonisins are difficult to detect by conventional methods, which result in an underestimation of their contamination level. Fumonisins widely contaminate maize and maize products, especially in broiler feed. As an economically important food, broilers are often adversely affected by mycotoxins, leading to food safety hazards and high economic losses. However, there are few studies regarding the adverse effects of FBs on broiler growth and health, especially modified FBs. Our data shows that after exposure to FBs or hydrolyzed fumonisin Bs (HFBs), the body weight and tissue weight of broilers decreased significantly, especially the testes. Moreover, they significantly affect the intestinal microbiota and the relative abundance of bacteria from phylum-to-species levels, with the differentially affected bacteria mainly belonging to *Firmicutes* and *Proteobacteria*. Our findings suggest that both the parent and hydrolyzed FBs could induce growth retardation, tissue damage and the imbalance of intestinal microbiota in broilers. This indicated that the harmful effects of HFBs cannot be ignored during food safety risk assessment.

## 1. Introduction

When maize is infected by the *Fusarium* species, it will not only reduce the yield and quality, but also result in the accumulation of toxic mycotoxins. Mycotoxins are synthesized in maize kernels and accumulate in maize-based feed directly [1,2]. The Food and Agriculture Organization (FAO) estimated that more than 25% of cereal crops are lost annually due to mycotoxin contamination [3]. In addition, mycotoxins can enter humans and animals through the food chain, which is a serious threat to human and animal health and causes heavy economic losses [4,5].

Fumonisin is a group of highly toxic low molecular weight mycotoxins, primarily produced by *Fusarium verticillioides* and *Fusarium proliferatum* that have been widely found to be contaminants worldwide [6,7]. To date, 28 chemical structures of fumonisin have been discovered. Among them, fumonisin B1 (FB1), fumonisin B2 (FB2) and fumonisin B3 (FB3) are the three main forms [8]. Fumonisin can be transformed into modified fumonisin, such as hydrolyzed fumonisin, after being metabolized by microorganisms or plants and even the grinding of grain samples. A survey of mycotoxin contamination of 4 batches of 327 grain samples worldwide showed that the positive rates of major fumonisin were approximately 58% (Africa), 51% (Central Europe), 27% (North America), 51% (Central Europe), 56% (South Asia) and 55% (Southeast Asia) in different areas [9]. Fumonisin mainly contaminate maize and maize products.

FB1 is the most polluted and toxic fumonisin. Studies have shown many adverse effects of fumonisin, including liver and kidney toxicity, enterotoxicity, immunosuppressive effects, neurodevelopmental toxicity, neonatal neural tube defects, and esophageal and liver cancer [10,11,12,13]. The International Agency for Research on Cancer (IRAC) listed fumonisin as a 2B carcinogen in 2002 [1]. However, multiple fumonisins are often found at the same time in the same environment. If only the toxicity of a single fumonisin is considered, and the interactions between mixtures (additive or synergistic or antagonistic effect) are ignored, the true toxicity of fumonisins cannot be accurately reflected in the actual environment [8,14]. Moreover, hydrolyzed fumonisin B1 (HFB1) was mentioned to competitively inhibit the enzyme ceramide synthase and to generate acetylated products [15]. However, the metabolism and toxicity mechanisms of modified fumonisin in animals have rarely been researched.

With its inherent advantages of high efficiency and low cost, the broiler breeding industry achieved a historical highest degree of industrialization in animal husbandry in China. Broiler production in China has increased from 1.358 million tons in 1984 to 11.44 million tons in 2009, and continues to grow at a rate of 5–10% per year. The Pudong Sanhuang broiler is a breed that is characteristic as one of the important economic animals in China with the advantages of a short growth cycle, strong disease resistance and rich nutritional value [16]. Most poultry broiler (early stage) feeds are severely contaminated by fumonisin. The contaminant rate of fumonisin B pollutants in compound feed and feed ingredients was 93.33% and 83.33%, respectively, and the highest feed detection level at 12.82 mg/kg was found in Korea in published papers [17]. In 2019, a survey of fumonisin contamination in maize feed in China showed that feed was not only contaminated by the parent fumonisin, but also approximately 71.23% of the samples were contaminated by modified fumonisin [3].

Based on this, this study used broiler chickens as an in vivo research model to explore the hazards of fumonisin Bs (FBs) and its modified forms to poultry. By using feed naturally contaminated with different levels of FBs (FB1, FB2 and FB3) or modified forms (without detectable levels of other mycotoxins), the effects of FBs on animal growth, blood biochemistry and intestinal microbiota can be observed. This study is useful to evaluate the health hazards and potential mechanisms of FBs, especially its modified forms in poultry.

## 2. Results

### 2.1. Effect of FBs and HFBs on Growth Performance in Broilers

To explore the effect of FBs and HFBs on the broilers, the death, body weight and feed intake of the broilers were monitored during the experiment. The weight of the broilers showed an upward trend, however, the weight of the broilers in the H-FBs (high-level fumonisin Bs) and H-HFBs (high-level hydrolyzed fumonisin Bs) groups was significantly lower than that in the other groups in the 6th week. As the feeding time increases, the differences between the groups becomes more evident (Table 1 and Table S1). The net weight of the broilers in the H-FBs group was significantly lower than that in the control and L-HFBs (low-level hydrolyzed fumonisin Bs) groups. The average weekly gain (AWG) of broilers in the H-FBs group was significantly lower than that in the L-FBs (low-level fumonisin Bs) and the L-HFBs groups. The feed intake in the treatment groups was lower than that in the control group. For the death of the broilers, it occurred in the second week, and the number of deaths was 0–3 per group (Table 2). In conclusion, FBs and HFBs could affect the growth performance of broilers, and H-FBs had a stronger influence, compared with H-HFBs.

### 2.2. Effect of FBs and HFBs on the Organ and Blood Index in Broilers

At the end of the experiment, the liver, kidney, and testis of the broilers were collected and weighed to explore the effect of the FBs and HFBs on the broiler organs. The FBs and HFBs had no effect on the kidney weight. Although the absolute weight of the liver in the treatment group decreased, the relative weight did not change significantly. Notably, the testis weight decreased significantly after FBs and HFBs treatment, suggesting that FBs and HFBs may influence the reproductive system in male broilers (Table 3).

FBs and HFBs have little effect on the blood index (Table 4). A glutamic-pyruvic transaminase (ALT) increase was observed in the L-FBs and H-HFBs groups, total bilirubin (TBIL) was decreased in the L-FBs and H-FBs groups, and no other abnormality was observed.

### 2.3. Effect of FBs and HFBs on the Intestinal Microbiota Composition

**Figure 1:**
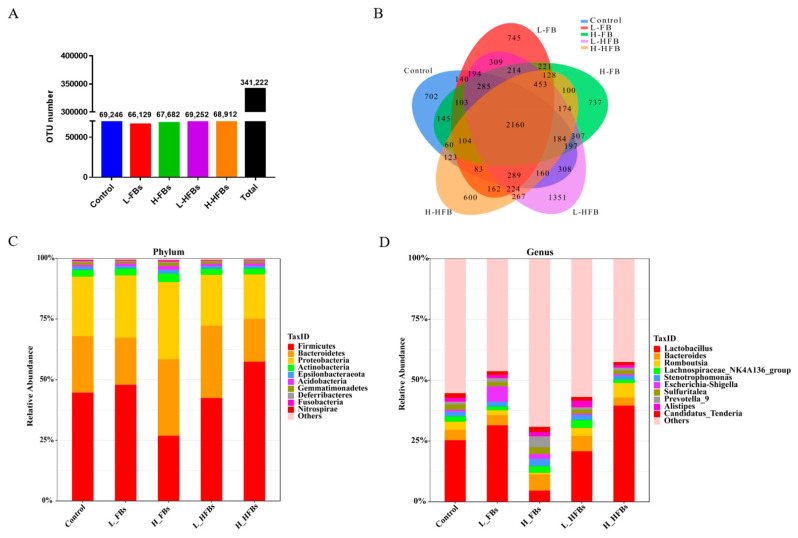
Fumonisin B and hydrolyzed fumonisin B alter the intestinal microbial composition. (**A**,**B**) OTUs and flower plot (the L-FBs was the low-level fumonisin Bs group, the H-FBs was the high-level fumonisin Bs group, the L-HFBs was the low-level hydrolyzed fumonisin Bs group and the H-HFBs was the high-level hydrolyzed fumonisin Bs group); the different colors and corresponding numbers represent the number of OTUs; (**C**) relative abundance of bacteria at the phylum level; (**D**) relative abundance of bacteria at the genus level.

To explore the effect of FBs and HFBs on the intestinal microbiota, the jejunum microbiota of the broilers was analyzed by 16S rRNA sequencing. All of the samples produced 341,222 OTUs (operational taxonomic units), and the number of OTUs in the five groups is similar (Figure 1A). The number of shared and unique OTUs among the five groups is shown in Figure 1B. There were 2160 shared OTUs between the control group and treatment group, and the numbers of unique OTUs in the control, L-FBs, H-FBs, L-HFBs and H-HFBs groups were 702, 745, 737, 1351 and 600, respectively.

Based on the OTUs, the relative abundance of intestinal microbiota was analyzed at the phylum-to-species levels. ANOVA and Kruskal–Wallis analysis were used for abundance comparison. More differentially expressed bacteria were detected by Kruskal–Wallis analysis, and the number of differentially expressed bacteria at the genus level is the largest (Table 5 and Table S2).

*Firmicutes*, *Bacteroidetes* and *Proteobacteria* are the three most abundant bacterial phyla in the broiler intestines (Figure 1C). Although the abundance of *Firmicutes* in the H-FBs group was relatively low and the abundance of *Bacteroidetes* in the H-FBs groups was relatively high compared with the other groups, there was no significant difference in the abundance of these dominant phyla due to the individual differences among the broilers. Only two bacterial phyla (*Elusimicrobia* and *Planctomycetes*) with significant differences were found by Kruskal–Wallis analysis, and no bacteria were found by ANOVA (Table S2).

The main classes of bacteria in the broilers are Bacilli, Bacteroidia and Clostridia (Figure S1A). Similar to the phyla, although the abundance of Bacilli in the H-FBs group is relatively low and the abundance of Bacteroidia and Clostridia is relatively high in the H-FBs and L-HFBs groups, there is no significant difference in the abundance of these dominant classes (Figure S1B). Using different analysis methods (ANOVA and Kruskal–Wallis), the common differences in the bacteria were found, including Elusimicrobia and Phycisphaerae (Table S2).

The main orders of bacteria in the broilers are Lactobacillales, Bacteroidales and Clostridiales (Figure S1C). Lactobacillales is significantly different between the H-FBs and L-FBs groups (Kruskal–Wallis analysis) (Figure S1D) and 7 common different bacteria are found (Table S2).

The main families of bacteria in the broilers are Lactobacillaceae, Muribaculaceae and Lachnospiraceae (Figure S1E). Lactobacillaceae is significantly decreased in the L-FBs groups (Kruskal–Wallis analysis) (Figure S1F) and 10 common different bacteria are found (Table S2).

At the genus level, the main bacteria in the broilers are Lactobacillus, Bacteroides and Romboutsia (Figure 1D), among which Lactobacillus is also significantly decreased in the L-FBs groups (Kruskal–Wallis analysis) and 21 common different bacteria are found by the 2 methods (Table S2).

Most of the bacteria belong to others at the species level and 7 common species are found (Figure S1G and Table S2).

### 2.4. Difference Analysis of Intestinal Microbiota after FBs and HFBs Treatment

The richness and diversity of the intestinal microbiota after FBs and HFBs treatment were assessed by alpha diversity analysis. Chao1 (Figure S2A) and ACE (Figure S2B) analyses were used to evaluate the richness, and Shannon (Figure S2C) and Simpson (Figure S2D) analyses were used to evaluate the diversity. Compared with the control group, FBs and HFBs had no effect on the richness and diversity of the intestinal microbiota of the broilers. There are significant differences in the intestinal microbiota diversity between the H-FBs and HFBs (Figure S2C,D), indicating that these two forms of mycotoxins have different effects on intestinal bacteria.

**Figure 2:**
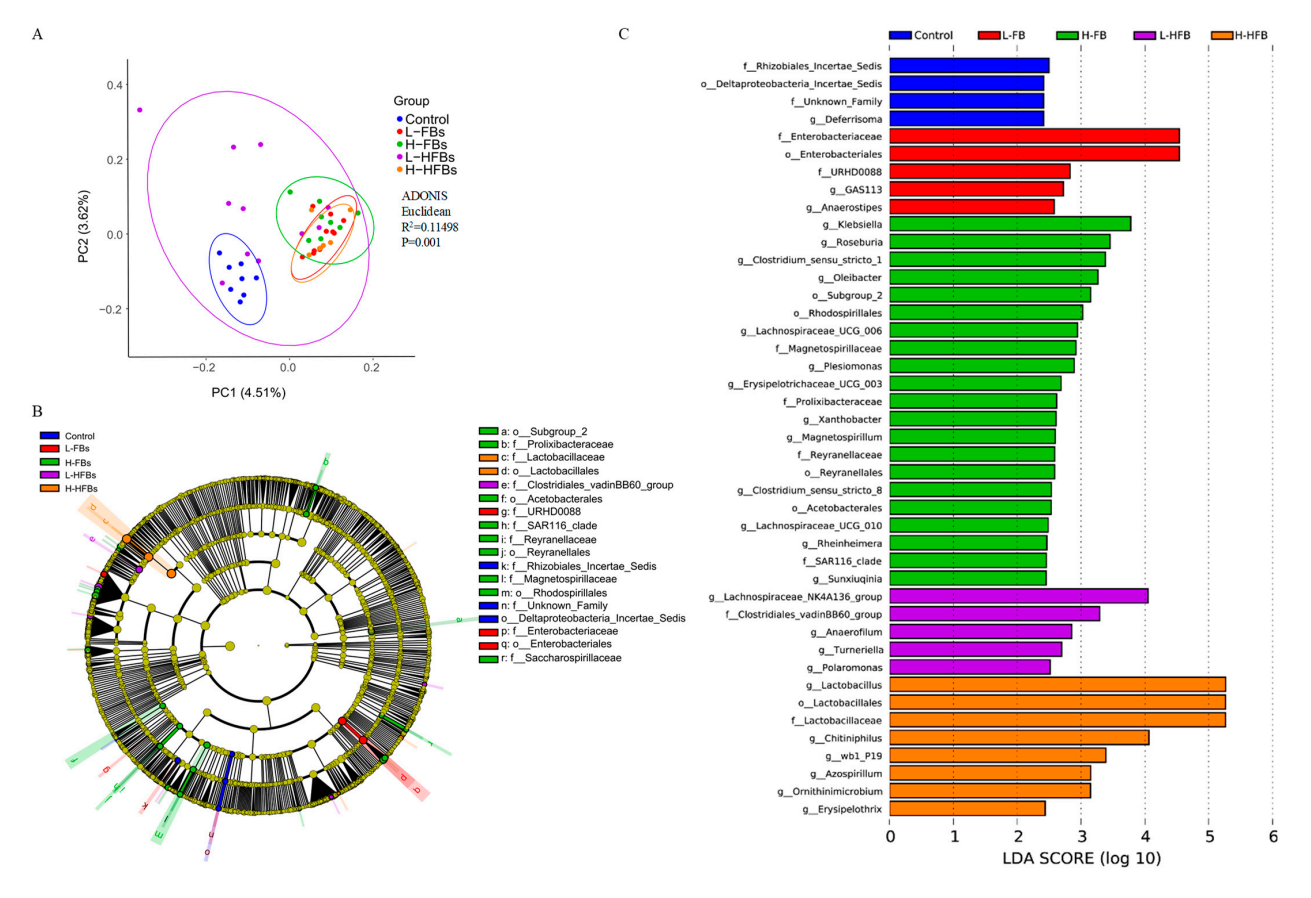
Difference analysis of intestinal microbiota after fumonisin B and hydrolyzed fumonisin B treatment. (**A**) PcoA analysis with binary_jaccard algorithm (the L-FBs was the low-level fumonisin Bs group, the H-FBs was the high-level fumonisin Bs group, the L-HFBs was the low-level hydrolyzed fumonisin Bs group and the H-HFBs was the high-level hydrolyzed fumonisin Bs group). (**B**) The enriched bacteria in each group generated from LEfSe analysis are represented in the cladogram. Each ring represents the phylum/class/order/family/genus from inside to outside. Different color nodes represent the bacteria with relatively high abundance and significant differences in the same color group, while yellow nodes represent the bacteria with no significant differences, and the diameter of each circle represents the relative abundance. (**C**) The most differentially abundant bacteria were identified through the LDA score. Different colors represent different groups. (o, order; f, family; g, genus).

Differences in each group were further analyzed by using beta diversity analysis. Principal coordinate analysis (PCoA) shows a clear separation between the control and treatment groups, except for the L-HFBs group, where the *p*-value of ADONIS is 0.001 (Figure 2A), demonstrating a strong effect of FBs and HFBs on the intestinal microbiota.

### 2.5. LEfSe Analysis of Intestinal Microbiota after FBs and HFBs Treatment

To explore the specific bacterial taxa associated with FBs and HFBs exposure, a linear discriminant analysis (LDA) coupled with an effect size measurements (LEfSe) comparison was performed. The predominant bacteria of the microbiota in each group are represented in the cladogram (Figure 2B). Although the results for most bacteria showed no difference, some specific bacteria were obtained, including 7 orders and 10 families. More predominant bacteria from the phylum-to-genus level were identified via the LDA score, and 26 genera were obtained (Figure 2C). Most of the specific taxa were from the two most dominant phyla, Proteobacteria (23) and Firmicutes (14). Most of the specific taxa were from the H-FBs, and the second most predominant group was H-HFBs, demonstrating a strong effect of H-FBs and H-HFBs on the intestinal microbiota.

At the genus level, the predominant bacteria in the L-FBs group are GAS113 and Anaerostipes (LDA > 2.5). The predominant bacteria in the H-FBs group are Klebsiella, Roseburia, Clostridium_sensu_stricto_1 and Oleibacter (LDA > 3). The predominant bacterium in the L-HFBs group is Lachnospiraceae_NK4A136_group (LDA > 4), and Lactobacillus (LDA > 5) and Chitiniphilus (LDA > 4) are the predominant bacteria in the H-HFBs group (Figure 2C).

**Figure 3:**
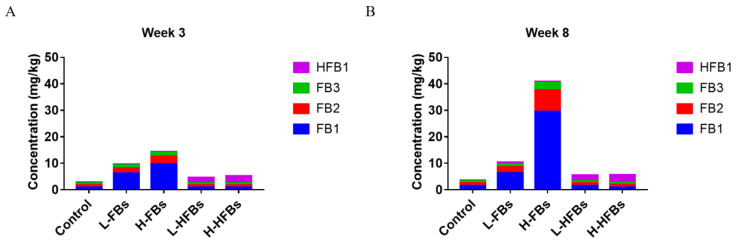
Fumonisin B and hydrolyzed fumonisin B residue in feces after 3 weeks (**A**) and 8 weeks (**B**) of feeding. The L-FBs was the low-level fumonisin Bs group, the H-FBs was the high-level fumonisin Bs group, the L-HFBs was the low-level hydrolyzed fumonisin Bs group and the H-HFBs was the high-level hydrolyzed fumonisin Bs group. The vertical height represents the content, and the different colors represent the different mycotoxins.

We further detected the content of FB1, FB2, FB3 and HFB1 in broiler feces (Figure 3). Compared with the control group, the content of FB1, FB2 and FB3 in the FBs group increased and was directly proportional to the concentration of FBs, while the content of HFB1 did not change. In the HFBs groups, the content of HFB1 increased, while the content of FBs did not change. These results proved that the toxicity effects mentioned above were caused by FBs and HFBs. Moreover, the content of FBs at 8 weeks was higher than that at 3 weeks, indicating that FBs have a significant cumulative effect.

## 3. Discussion

With the development of the broiler industry, the per capita consumption of broilers far exceeds that of pork and beef. However, since the main ingredient of broiler feed is maize, the health of broiler chickens is threatened by mycotoxins, especially fumonisin, which mainly pollutes maize. Broilers fed with a mycotoxin-contaminated feed will not only experience adverse effects on their growth and development, but also cause serious economic losses. At the same time, mycotoxins will accumulate in the edible parts of the broilers and spread to humans through the food chain, posing a threat to human health. Therefore, in this study, the health hazards of FBs and HFBs to broilers were explored, laying a foundation for the establishment of methods to prevent and avoid health hazards in the future.

Many studies have shown that fumonisin could significantly reduce the growth performance of pigs, such as body weight and food intake [18]. Similarly, the body weight and food intake of broilers were significantly reduced by FBs in our study. The same phenomenon also occurred in the HFBs treatment group. Similar to the results of the previous study, hydrolyzed fumonisin was less toxic than fumonisin, but still had a significant difference compared to the control group [19]. In addition to reduced growth performance, there were no obvious external clinical symptoms of fumonisin poisoning, such as coughing. Although maize contaminated with mycotoxins may have a reduced oil content, chemical analysis of the diet showed that there was no difference in the main nutritional value between treatments [20]. Therefore, the decline in growth performance is probably not caused by the difference in nutritional value after mycotoxin contamination, but may be caused by harmful effects after FBs poisoning [21]. Broiler death occurred in both the control group and the experimental group, and there was no significant difference between the groups, indicating that the death of broilers was not related to fumonisin exposure. This is consistent with the results of previous studies; 20 mg/kg FB1 + FB2 does not cause death in poultry and pigs [22,23].

The liver and kidney are the main target organ for fumonisin toxicity. Many studies have shown that fumonisin can induce liver and kidney cell damage in a variety of ways, such as oxidative stress, endoplasmic reticulum stress and autophagy [24]. Andras found that 10 days after exposure to FB1, the liver showed pathological changes in rats [25]. Another study also showed that FB1 could significantly affect liver fatty acid metabolism [26]. We found that both FBs and HFBs could significantly reduce the liver weight and increase the level of ALT in the serum. However, the weight of the kidney and the content of CREA1, UA and BUN in the serum did not change significantly. This phenomenon is consistent with those reported in the literature [23]. Surprisingly, the weight of the testis was significantly reduced after the emergence of FBs and HFBs, which means that the adverse effects of FBs and HFBs on the male reproductive system are worthy of attention in the future.

The microbiota in animal intestines plays an important role in the health of the host, which is a widely accepted fact [18,27]. Many immune and metabolic diseases, such as chronic inflammation, obesity, diabetes, inflammatory bowel disease and atherosclerosis, are likely to be related to the imbalance of intestinal microbiota [28,29,30]. Increasing attention has been given to the balance of intestinal microbiota [31,32]. Researchers found that fumonisin could alter the intestinal barrier in broilers [33]. Additionally, Zhang and co-researchers reported the response of the fecal bacterial microbiota to FB1 exposure in BALB/c mice [34]. However, there are few studies on the effect of fumonisin on the intestinal microbiota of animals, such as weaned pigs and broilers. In this study, FBs and HFBs affected the intestinal microbiota of broilers, particularly the H-FBs.

The three predominant bacterial phyla in this study were *Firmicutes*, *Bacteroidetes* and *Proteobacteria*. is the predominant Gram-negative bacterial phylum in the gastrointestinal tract, and is a major participant in the healthy state and complex homeostasis protected by the gut microbiota. An abnormal distribution of *Bacteroides* could cause growth retardation, immune disorders and metabolic disorders [35,36]. *Bacteroides* and *Firmicutes* are also related to obesity. A higher quantity of *Firmicutes* in the intestine leads to a more effective absorption of calories from food, possibly causing obesity. Studies have shown that the number of *Firmicutes* in the intestine of obese mice is higher than that of *Bacteroides*. *Bacteroides* have beneficial effects on body weight gain and insulin sensitivity in high-fat diet-induced obese mice [37,38,39]. When pigs were fed wheat contaminated with deoxynivalenol (DON), the abundance levels of *Firmicutes* and *Bacteroides* in the cecum, colon and ileum changed [40]. In our results, there was no difference in *Bacteroidetes*, *Proteobacteria* or *Firmicutes* among the different groups due to individual differences, but the ratio of *Firmicutes/Bacteroidetes* was lower in the H-FBs group (the *p*-value between L-FBs and H-FBs was 0.0896), which may explain the weight loss of the broilers. These results confirm the results obtained in previous studies. *Proteobacteria* are spoilage bacteria and often appear in the intestines of humans and animals. When the intestines are exposed to contaminants, *Proteobacteria* increase significantly [41]. *Proteobacteria* can affect the function of the gastrointestinal tract and cause many diseases [42]. After fumonisin exposure, *fusobacteria* also appeared on the list of dominant strains, which can cause mucosal infections and enteritis [43]. These gut-damaging factors can affect the absorption of nutrients, leading to poor broiler growth.

LEfSe analysis indicated that most of the differential species belonged to *Proteobacteria* and *Firmicutes*. However, each treatment group mainly affected different microorganisms. The FBs group mainly affected *Klebsiella* and *Anaerostipes*. As pathogenic microorganisms, they could infect many organs and cause functional damage, including to the intestinal tract [44,45]. The HFBs group mainly affected *Lachnospiraceae_NK4A136_group* and *Chitiniphilus*. Interestingly, a kind of beneficial bacteria, *Lactobacillus,* was lower in the H-FBs group, while it was in relatively high abundance in the H-HFBs group, indicating a difference between H-FBs and H-HFBs.

Mycotoxin was mainly excreted through the intestine to the feces [46]. The excessive accumulation of mycotoxin may lead to the destruction of the intestinal microbial balance. Chang and co-workers reported that when aflatoxin and zearalenone were degraded by microorganisms (*Bacillus subtilis*, *Lactobacillus casei* and *Candida utilis*), the abnormal intestinal microbes caused by aflatoxin and zearalenone in broilers were significantly alleviated [47]. Li and co-workers also found that the application of *Clostridium* sp. *WJ06* can reduce the toxic effects of DON and recover the intestinal microecosystem of growing pigs [40]. Our results show that as the treatment time increases, the fumonisin level in the feces shows an increasing trend. The imbalance of intestinal microbials may be caused by excessive fumonisin accumulated in the intestine. Furthermore, the bioavailability of HFB1 in rats is greater than that of FB1 [15]. Our results also reveal that even if the exposure dose of fumonisin and the modified form are the same, the level of toxins in the feces of the HFBs group is much lower than that in the FBs group, which means that the modified fumonisin may have higher bioavailability or be easily transformed, making itself difficult to be detected by current approaches.

## 4. Conclusions

In conclusion, more attention should be focused on modified mycotoxin and toxin mixtures during the security risk assessment. When broilers were fed with naturally fumonisin-contaminated maize for 8 weeks, the growth performance of broilers was adversely affected by fumonisins. Fumonisins could significantly decrease body weight and some tissue weight, especially the testes. This indicated that the male reproductive system may be more susceptible to fumonisins. At the same time, the fumonisin level in the feces was significantly increased, and their intestinal microbiota balance was significantly changed, which may be one of the reasons directly affecting their body weight loss. Hydrolyzed fumonisin, which is a common modified fumonisin, can also cause the same adverse effects, although the degree is lower than that of the parent fumonisin, which indicates that modified fumonisins also have high safety risks. In the future, the hazards of modified fumonisins in food safety risk assessment should be considered. Finally, this study facilitates the determination or optimization of the legal limits of mycotoxins in food and feed.

## 5. Materials and Methods

### 5.1. Chemicals and Materials

FB1, B2 and B3 were purchased from Abcam (Cambridge, MA, USA). HFB1 was obtained from Romer. Ultrapure water (18.2 MΩ·cm) was supplied by Millipore (Bedford, MA, USA). Acetonitrile and methanol (HPLC grade) were purchased from Honeywell (Morristown, NJ, USA). Formic acid (HPLC grade) was obtained from Anpel (Shanghai, China). Cleanert MC clean-up columns were obtained from Bonna-Agela Technologies (Tianjin, China). Maize grains were purchased from a local market in Shanghai, China. Enzyme-linked immunosorbent assay (ELISA) kits were purchased from Wako (Kanto, Japan). Potato dextrose agar medium (PDA) and blood collection tubes were purchased from BD Difco (San Diego, CA, USA).

### 5.2. Experimental Animals and Feeding

Sixty male one-day-old Pudong Sanhuang broiler chickens were obtained from Shanghai Veterinary Research Institute, Chinese Academy of Agricultural Sciences (Shanghai, China). The experiment was approved by the Welfare and Ethics Committee of Experimental Animals in Shanghai Veterinary Research Institute, Chinese Academy of Agricultural Sciences (Shanghai, China). The Ethical approval code was SV-20200906-Y06. Additionally, the Ethical approval date was 6 September 2020. Animal experiments followed the National Research Council’s Guide for the Care and Use of Laboratory Animals.

The broilers were randomly divided into five groups: control group, L-FBs group (low-level fumonisin Bs), H-FBs (high-level fumonisin Bs), L-HFBs (low-level hydrolyzed fumonisin Bs) and H-HFBs (high-level hydrolyzed fumonisin Bs) groups, with 12 replicates per group. After a 1-week observation period, the experiment was conducted for 8 weeks after changing to the mycotoxin-containing feed. Animals were housed in stainless steel cages with free access to water and food. Animal body weight and feed consumption were recorded during the experiment.

### 5.3. Preparation of the Experimental Feed

*F. verticillioides BJ6* was isolated by our laboratory. Additionally, the strain was maintained as spore suspensions in 20% glycerol at −80 °C. *F. verticillioides BJ6* was inoculated into PDA medium and cultured at 25 °C for 7 days. Maize grains were irradiated with a cobalt radiation source (8–10 kGy) for sterilization and then put into conical flask and rehydrated to water activity (aw) 0.99 by the addition of sterile distilled water as a maize culture medium. Due to a lack of relevant standards, FB1, FB2, FB3 and HFB1 were detected, and the different groups were:

Control group: maize culture medium was inoculated with blank PDA medium, cultured at 25 °C for 10 days, crushed and mixed evenly with ordinary broiler feed. The diet composition in the ordinary broiler feed is shown in Table 6. The concentration of FB1, FB2, FB3 and HFB1 was 74.10 μg/kg, 15.93 μg/kg, 12.16 μg/kg and 7.75 μg/kg in the ordinary broiler feed, respectively.

FBs group: maize culture medium was inoculated with *F. verticillioides BJ6* colonies taken from the edges of old colony-edge bacteria, cultured at 25 °C for 10 days, crushed and mixed evenly with ordinary broiler feed. The concentration of FBs was 10 mg/kg (FB1 + FB2 + FB3) in the L-FBs group and 20 mg/kg (FB1 + FB2 + FB3) in the H-FBs group.

HFBs group: maize meal containing FBs was converted into HFBs through alkaline hydrolysis (all FBs disappeared) and mixed evenly with ordinary broiler feed. The doses were the same as the FBs group.

### 5.4. Mycotoxins Extraction

Samples were dried at 65 °C and milled into 0.45 mm flour. Briefly, 1 ± 0.05 g samples were extracted by 10 mL extracting solution (acetonitrile:water:formic acid = 840:159:1, *v/v*). Samples were shaken at 2500 rpm/min in an orbital shaker for 20 min and then ultrasonicated for 30 min. Then, they were centrifuged at 4000 rpm/min for 10 min. Cleanert MC columns were used to purify the supernatant. One milliliter of purified supernatant was filtered through a 0.22 μm nylon filter and stored in sampler vials at −20 °C until high-performance liquid chromatography-mass spectrometry analysis, and the analysis method was the same as that described in a previous article [3].

### 5.5. Collection and Analysis of Blood and Tissue Samples

After 8 weeks of feeding, the broilers were sacrificed by exsanguination from the jugular vein after taking a blood sample from the wing vein. Blood was stored in procoagulant tubes and anticoagulant tubes. Serum samples were separated by centrifugation (Thermo, Waltham, MA, USA) of the blood at 1200× *g* for 10 min at 4 °C. All of the samples were temporarily stored at 4 °C and tested within a day. The serum samples were analyzed using ELISA kits (Wako, Tokyo, Japan) according to the manufacturer’s instructions and detected in an automatic biochemical analyzer (HITACHI, Tokyo, Japan). Hemograms were generated using a BC-3800 Automated Hematology Analyzer (Shenzhen, China). For conventional analysis, the liver, kidney, and testicles were collected and weighed.

### 5.6. Sequencing, Data Processing and Analysis of 16S rRNA Amplicons of Intestinal Bacteria

Sequencing and preliminary data processing were conducted by OE Biotech Co., Ltd. (Shanghai, China). Total genomic DNA was extracted from the jejunum contents using a DNA Extraction Kit (Magen, Guangzhou, China) following the manufacturer’s instructions. The concentration of DNA was verified with a NanoDrop2100 (Thermo, Waltham, MA, USA) and agarose gel electrophoresis. The genomic DNA was used as a template for PCR (V3–V4 variable regions of the bacterial 16S rRNA genes) amplification with the primers 343F (5′-TAC GGR AGG CAG CAG-3′) and 798R (5′-AGG GTA TCT AAT CCT-3′) and Tks Gflex DNA Polymerase (Takara, Tokyo, Japan)). The first PCR reactions were conducted using the following program: 5 min of pre-degeneration at 94 °C, 26 cycles of 30 s for degeneration at 94 °C, 30 s for annealing at 56 °C, 20 s for elongation at 72 °C, and a final extension at 72 °C for 5 min. The samples were stored at 4 °C after the reaction. The amplicon quality was visualized using gel electrophoresis, purified with AMPure XP beads according to the manufacturer’s instructions (Agencourt, San Diego, CA, USA), and amplified for another round of PCR. The second PCR reactions were conducted using the following program: 5 min of pre-degeneration at 94 °C, 7 cycles of 30 s for degeneration at 94 °C, 30 s for annealing at 56 °C, 20 s for elongation at 72 °C, and a final extension at 72 °C for 5 min. The samples were stored at 4 °C after the reaction. After purification with AMPure XP beads, the final amplicon was quantified using a Qubit dsDNA assay kit (Life Technologies, Waltham, MA, USA). Equal amounts of purified amplicons were pooled for subsequent sequencing.

Paired-end reads of raw fastq files were preprocessed using Trimmomatic software [48] with the following parameters: (1) ambiguous bases (N) were cut off, and (2) low-quality sequences with an average quality score below 20 were cut off using a sliding window trimming approach. After trimming, paired-end reads were assembled using FLASH software [49] with the following parameters: (1) minimal overlapping was 10 bp; (2) maximum overlapping was 200 bp; and (3) the maximum mismatch rate% was 20%. The sequences were further denoised and the reads were removed with chimeras using QIIME software (version 1.8.0) [50] to produce clean reads. Then, the clean reads were subjected to primer sequence removal and clustering to generate operational taxonomic units (OTUs) using Vsearch software (Rognes et al., 2016) with a 97% similarity cut-off. The representative read of each OTU was selected using the QIIME package and annotated and blasted against the Silva database (Version 138) using the RDP classifier [51] (the confidence threshold was 70%).

Data was uploaded to National Center for Biotechnology Information which can be downloaded by using BioProject ID: PRJNA784726.

### 5.7. Statistical Analysis

The data were analyzed by one-way ANOVA with the Tukey’s multiple comparisons test (parametric test) or Kruskal–Wallis test with Dunn’s multiple comparisons test (non-parametric test) to assess the differences between the groups using the GraphPad Prism 7.00, and the values are presented as the mean ± standard deviation (SD). *p* < 0.05 was considered statistically significant, and the level of significance in this manuscript was set at *, *p* < 0.0332; **, *p* < 0.0021; ***, *p* < 0.0002; ****, *p* < 0.0001.

## Figures and Tables

**Table 1:** Body weight of broilers after fumonisin B and hydrolyzed fumonisin B treatment.

Weeks	Groups ^1^				
	Control	L-FBs	H-FBs	L-HFBs	H-HFBs
0	90.79 ± 6.47 ^2^	87.17 ± 7.04	90.22 ± 11.53	94.23 ± 6.97	91.89 ± 7.86
1	223.17 ± 16.54	228.75 ± 21	217.92 ± 22.44	236.67 ± 13.72	221.92 ± 17.71
2	389.33 ± 36.57	385.25 ± 47.85	368.73 ± 38.17	374.42 ± 39.43	356.27 ± 37.73
3	592.3 ± 41.4	553.18 ± 68.44	563.22 ± 67.32	584.58 ± 60.65	553.22 ± 43.18
4	854.2 ± 99.06	787.64 ± 97.08	801.11 ± 113.09	846 ± 61	818.89 ± 61.95
5	1112 ± 98.31	1014.09 ± 88.43	990 ± 160.97	1028.75 ± 112.56	1011.11 ± 82.79
6	1425 ± 118.01	1470 ± 125.5	1290 ± 194.88	1438.75 ± 158.44	1292.22 ± 183.13
7	1604 ± 111.53	1541 ± 110.76	1355.56 ± 218.34 *** ^3^	1546.58 ± 156.92	1370 ± 258.52 ***
8	1796 ± 127.83	1740 ± 134.38	1462.22 ± 213.98 ****	1766.25 ± 191.63	1511.11 ± 330.37 ****

^1^ The L-FBs was low-level fumonisin Bs group, the H-FBs was high-level fumonisin Bs group, the L-HFBs was low-level hydrolyzed fumonisin Bs group and the H-HFBs was high-level hydrolyzed fumonisin Bs group. ^2^ Data are expressed as the mean ± SD. ^3^ Data are analyzed by the one-way ANOVA with Tukey’s multiple comparisons test (parametric test, *** *p* < 0.0002, **** *p* < 0.0001).

**Table 2:** Fumonisin B and hydrolyzed fumonisin B impair the growth performance of broilers.

	Control	L-FBs ^1^	H-FBs	L-HFBs	H-HFBs
Net weight (kg)	1.71 ± 0.14 ^2^	1.65 ± 0.14	1.37 ± 0.22 * ^3^	1.67 ± 0.20	1.42 ± 0.35
AWG (g/b/w)	199.02 ± 36.27	208.18 ± 29.89	160.55 ± 31.61	209.00 ± 24.85	173.63 ± 45.67
Feed intake (kg)	60.1	51.3	39.7	50.3	41.8
Mortality (%)	16.67	16.67	25	0	25

^1^ The L-FBs was low-level fumonisin Bs group, the H-FBs was high-level fumonisin Bs group, the L-HFBs was low-level hydrolyzed fumonisin Bs group and the H-HFBs was high-level hydrolyzed fumonisin Bs group. ^2^ Data are expressed as the mean ± SD. ^3^ Data are analyzed by the one-way ANOVA with Tukey’s multiple comparisons test (parametric test, * *p* < 0.0332).

**Table 3:** Effect of fumonisin B and hydrolyzed fumonisin B on tissues in broilers.

	Control	L-FBs ^1^		H-FBs	L-HFBs	H-HFBs
Liver (g)	35.62 ± 3.45 ^2^	32.86 ± 4.18		23.18 ± 3.12 *** ^3^	30.82 ± 4.52	25.6 ± 10.61 *
Kidney (g)	12.74 ± 1.75	11.23 ± 1.82		9.61 ± 1.6	10.81 ± 2.81	9.68 ± 3.18
Testis (g)	2.55 ± 1.77	1.05 ± 0.62 *		0.87 ± 0.43 *	1.46 ± 0.85	0.88 ± 0.82 *
Liver/weight (g/kg)	19.73 ± 1.58	18.9 ± 2.52		15.93 ± 1.18	17.63 ± 3.57	16.46 ± 5.14
Kidney/weight (g/kg)	7.11 ± 0.95	6.42 ± 0.74		6.58 ± 0.45	6.18 ± 1.47	6.25 ± 0.92
Testis/weight (g/kg)	1.43 ± 1	0.58 ± 0.32 *		0.57 ± 0.19 *	0.81 ± 0.47	0.55 ± 0.43 *

^1^ The L-FBs was low-level fumonisin Bs group, the H-FBs was high-level fumonisin Bs group, the L-HFBs was low-level hydrolyzed fumonisin Bs group and the H-HFBs was high-level hydrolyzed fumonisin Bs group. ^2^ Data are expressed as the mean ± SD. ^3^ Data are analyzed by the one-way ANOVA with Tukey’s multiple comparisons test (parametric test, * *p* < 0.0332, *** *p* < 0.0002).

**Table 4:** Effect of FBs and HFBs on blood index in the broiler.

Index	Units	Control	L-FBs ^1^	H-FBs	L-HFBs	H-HFBs
**Serum**						
ALT	U/L	1.4 ± 0.491 ^2^	2.11 ± 0.31 * ^3^	1.89 ± 0.57	1.91 ± 0.29	2.13 ± 0.6 *
AST	U/L	214.7 ± 25.1	197.89 ± 28.2	213 ± 34.69	196.36 ± 14.74	206.63 ± 34.82
ALB	g/L	17.74 ± 1.58	15.79 ± 1.73	17.39 ± 2.59	16.18 ± 2.15	16.71 ± 2.45
TBIL	μmol/L	5.88 ± 1.11	3.83 ± 1.55 **	3.11 ± 1.07 ***	4.45 ± 1.09	5.28 ± 0.77
ALP	U/L	1143.2 ± 330.72	891.78 ± 217.14	968.56 ± 318.26	849.45 ± 234.98	920.13 ± 361.36
BUN	mmol/L	1.14 ± 0.21	1.11 ± 0.34	1.15 ± 0.41	1.09 ± 0.12	1.11 ± 0.2
UA	μmol/L	232.63 ± 90.78	295.07 ± 138.13	225.81 ± 92.64	261.37 ± 101.33	274.83 ± 83.41
TG	mmol/L	0.45 ± 0.21	0.5 ± 0.27	0.31 ± 0.35	0.26 ± 0.12	0.35 ± 0.25
CREA1	μmol/L	7.83 ± 1.69	8.42 ± 2.21	6.98 ± 2.54	9.55 ± 3.09	7.76 ± 2.02
**Plasma**						
WBC	1 × 10^9^/L	244.52 ± 11.22	236.71 ± 10.02	238.63 ± 9.77	247.28 ± 9.86	224.11 ± 76.77
RBC	1 × 10^12^/L	2.99 ± 0.5	2.72 ± 0.41	2.84 ± 0.26	2.95 ± 0.35	3 ± 1.15
HGB	g/L	178.56 ± 27.1	164.13 ± 22.75	167 ± 11.73	178.89 ± 23.54	180.62 ± 67.9
HCT	%	39.92 ± 6.49	35.53 ± 5.08	36.61 ± 3.19	38.6 ± 5.01	39.61 ± 15.03
MCV	fL	133.73 ± 2.17	131.26 ± 5.14	129.09 ± 1.08	130.88 ± 5.87	118.27 ± 39.92
MCH	pg	59.82 ± 1.59	60.54 ± 2.07	58.91 ± 2.55	60.49 ± 1.42	53.69 ± 18.57
MCHC	g/L	447.78 ± 10.88	461.88 ± 5.71	456.75 ± 18.92	463.44 ± 18.9	407.32 ± 138.18
RDW	%	8.83 ± 0.54	8.49 ± 0.35	8.34 ± 0.3	8.82 ± 0.68	7.72 ± 2.53
PLT	1 × 10^9^/L	21.78 ± 8.51	18 ± 5.41	24.25 ± 13.3	30.11 ± 9.22	23.25 ± 8.66
MPV	fL	5.28 ± 0.28	5.28 ± 0.41	4.98 ± 0.35	5.18 ± 0.36	4.66 ± 1.61
PDW		16.96 ± 0.51	17.44 ± 1.07	16.63 ± 0.37	17.01 ± 0.61	14.93 ± 5.09
PCT	%	0.0112 ± 0.0046	0.0115 ± 0.0056	0.0116 ± 0.0066	0.015 ± 0.0048	0.012 ± 0.0057

^1^ The L-FBs was low-level fumonisin Bs group, the H-FBs was high-level fumonisin Bs group, the L-HFBs was low-level hydrolyzed fumonisin Bs group and the H-HFBs was high-level hydrolyzed fumonisin Bs group. ^2^ Data are expressed as the mean ± SD. ^3^ Data are analyzed by the one-way ANOVA with Tukey’s multiple comparisons test (parametric test, * *p* < 0.0332, ** *p* < 0.0021, *** *p* < 0.0002).

**Table 5:** Differential enrichment bacterial analysis.

Method	OTU	Phylum	Class	Order	Family	Genus	Species
ANOVA	256	0	2	12	15	34	10
Kruskal–Wallis	423	2	3	17	27	66	25

**Table 6:** The diet composition of the ordinary broiler feed.

Composition	Content (%)
Crude protein	19
Crude fiber	5
Crude ash	8
Calcium	0.7
Phosphorus	0.5
Sodium	0.3
Lysine	0.9
Methionine	0.35

## Data Availability

The data that support the findings of this study are available from the corresponding author upon reasonable request.

## Supplementary Materials

The following supporting information can be downloaded at https://www.mdpi.com/article/10.3390/toxins14030163/s1, Figure S1: Relative abundance of bacteria and analysis of dominant bacteria at class (A,B), order (C,D), family (E,F) and species (G) level; Figure S2: Alpha analysis of intestinal microbiota after FBs and HFBs treatment. (A) chao 1 analysis. (B) ACE analysis. (C) Shannon analysis. (D) Simpson analysis; Table S1: FBs and HFBs affect the body weight of broilers; Table S2: Differentially expressed bacteria after FBs and HFBs treatment.

## Abbreviations

F. verticillioides	Fusarium verticillioides
FBs	Fumonisin Bs
FB1	Fumonisin B1
FB2	Fumonisin B2
FB3	Fumonisin B3
HFBs	Hydrolyzed Fumonisin Bs
HFB1	Hydrolyzed Fumonisin B1
L-FBs	Low-level fumonisin Bs
H-FBs	High-level fumonisin Bs
L-HFBs	Low-level hydrolyzed fumonisin Bs
H-HFBs	High-level hydrolyzed fumonisin Bs
PDA	Potato dextrose agar medium
a_w_	Water activity
ALT	Alanine aminotransferase
AST	Aspartate aminotransferase
ALB	Albumin
TBIL	Total bilirubin
ALP	Alkaline phosphatase
BUN	Urea nitrogen
UA	Uric acid
TG	Triglycerides
CREA1	Creatinine 1
ADG	Average daily gain
ADFI	Average daily feed intake
F/G	Feed/gain
OUT	Operational taxonomic unit
PcoA	Principal coordinate analysis
LDA	Linear discriminant analysis
LEfEe	LDA coupled with effect size measurements
Don	Deoxynivalenol
ELISA	Enzyme-linked immunosorbent assay

## References

[1] Cao C., Zhu X., Li X., Ouyang H., Wang K., Li X. (2020). Assessment of ionic homeostasis imbalance and cytochrome P450 system disturbance in mice during fumonisin B1 (FB1) exposure. Chemosphere.

[2] Latorre A., Dagnac T., Lorenzo B.F., Llompart M. (2015). Occurrence and stability of masked fumonisins in corn silage samples. Food Chem..

[3] Yu S., Jia B., Li K., Zhou H., Lai W., Tang Y., Yan Z., Sun W., Liu N., Yu D. (2021). Pre-warning of abiotic factors in maize required for potential contamination of fusarium mycotoxins via response surface analysis. Food Control.

[4] Li X., Cao C., Zhu X., Li X., Wang K. (2020). Fumonisins B1 exposure triggers intestinal tract injury via activating nuclear xenobiotic receptors and attracting inflammation response. Environ. Pollut..

[5] Van der Westhuizen L., Shephard G.S., Burger H.M., Rheeder J.P., Gelderblom W.C., Wild C.P., Gong Y.Y. (2011). Fumonisin B1 as a urinary biomarker of exposure in a maize intervention study among South African subsistence farmers. Cancer Epidemiol. Biomark. Prev..

[6] Cendoya E., Chiotta M.L., Zachetti V., Chulze S.N., Ramirez M.L. (2018). Fumonisins and fumonisin-producing Fusarium occurrence in wheat and wheat by products: A review. J. Cereal Sci..

[7] Chen C., Mitchell N.J., Gratz J., Houpt E.R., Gong Y., Egner P.A., Groopman J.D., Riley R.T., Showker J.L., Svensen E. (2018). Exposure to aflatoxin and fumonisin in children at risk for growth impairment in rural Tanzania. Environ. Int..

[8] Yu S., Jia B., Liu N., Yu D., Wu A. (2020). Evaluation of the Individual and Combined Toxicity of Fumonisin Mycotoxins in Human Gastric Epithelial Cells. Int. J. Mol. Sci..

[9] Yu S., Jia B., Yang Y., Liu N., Wu A. (2020). Involvement of PERK-CHOP pathway in fumonisin B1-induced cytotoxicity in human gastric epithelial cells. Food Chem. Toxicol..

[10] Li Y., Fan Y., Xia B., Xiao Q., Wang Q., Sun W., Zhang H., He C. (2017). The immunosuppressive characteristics of FB1 by inhibition of maturation and function of BMDCs. Int. Immunopharmacol..

[11] Lin Y., Totsuka Y., Shan B., Wang C., Wei W., Qiao Y., Kikuchi S., Inoue M., Tanaka H., He Y. (2017). Esophageal cancer in high-risk areas of China: Research progress and challenges. Ann. Epidem..

[12] Liu X., Fan L., Yin S., Chen H., Hu H. (2019). Molecular mechanisms of fumonisin B1-induced toxicities and its applications in the mechanism-based interventions. Toxicon.

[13] Bouhet S., Oswald I.P. (2007). The intestine as a possible target for fumonisin toxicity. Mol. Nutr. Food Res..

[14] Yang Y., Yu S., Tan Y., Liu N., Wu A. (2017). Individual and Combined Cytotoxic Effects of Co-Occurring Deoxynivalenol Family Mycotoxins on Human Gastric Epithelial Cells. Toxins.

[15] Seiferlein M., Humpf H.U., Voss K.A., Sullards M.C., Allegood J.C., Wang E., Merrill A.H. (2007). Hydrolyzed fumonisins HFB1 and HFB2 are acylated in vitro and in vivo by ceramide synthase to form cytotoxic N-acyl-metabolites. Mol. Nutr. Food Res..

[16] Wu B., Cui H., Peng X., Fang J., Zuo Z., Deng J., Huang J. (2014). Dietary nickel chloride induces oxidative stress, apoptosis and alters Bax/Bcl-2 and caspase-3 mRNA expression in the cecal tonsil of broilers. Food Chem. Toxicol..

[17] Kim D.H., Lee I.H., Do W.H., Nam W.S., Li H., Jang H.S., Lee C. (2013). Incidence and levels of deoxynivalenol, fumonisins and zearalenone contaminants in animal feeds used in Korea in 2012. Toxins.

[18] Meerpoel C., Vidal A., Huybrechts B., Tangni E.K., De Saeger S., Croubels S., Devreese M. (2020). Comprehensive toxicokinetic analysis reveals major interspecies differences in absorption, distribution and elimination of citrinin in pigs and broiler chickens. Food Chem. Toxicol..

[19] Gu M.J., Han S.E., Hwang K., Mayer E., Reisinger N., Schatzmayr D., Park B.C., Han S.H., Yun C.H. (2019). Hydrolyzed fumonisin B1 induces less inflammatory responses than fumonisin B1 in the co-culture model of porcine intestinal epithelial and immune cells. Toxicol. Lett..

[20] Bartov I. (1985). Comparative effects of antifungal compounds on the nutritional value of diets containing moldy corn for broiler chicks. Poult. Sci..

[21] Rao Z.X., Tokach M.D., Woodworth J.C., DeRouchey J.M., Goodband R.D., Calderon H.I., Dritz S.S. (2020). Effects of Fumonisin-Contaminated Corn on Growth Performance of 9 to 28 kg Nursery Pigs. Toxins.

[22] Metayer J.-P., Travel A., Mika A., Bailly J.-D., Cleva D., Boissieu C., Guennec J.L., Froment P., Albaric O., Labrut S. (2019). Lack of Toxic Interaction between Fusariotoxins in Broiler Chickens Fed throughout Their Life at the Highest Level Tolerated in the European Union. Toxins.

[23] Murugesan G.R., Ledoux D.R., Naehrer K., Berthiller F., Applegate T.J., Grenier B., Phillips T.D., Schatzmayr G. (2015). Prevalence and effects of mycotoxins on poultry health and performance, and recent development in mycotoxin counteracting strategies. Poult. Sci..

[24] Liu X., Zhang E., Yin S., Zhao C., Fan L., Hu H. (2020). Activation of the IRE1alpha Arm, but not the PERK Arm, of the Unfolded Protein Response Contributes to Fumonisin B1-Induced Hepatotoxicity. Toxins.

[25] Szabo A., Szabo-Fodor J., Kachlek M., Mezes M., Balogh K., Glavits R., Ali O., Zeebone Y.Y., Kovacs M. (2018). Dose and Exposure Time-Dependent Renal and Hepatic Effects of Intraperitoneally Administered Fumonisin B(1) in Rats. Toxins.

[26] Ali O., Szabo-Fodor J., Febel H., Mezes M., Balogh K., Glavits R., Kovacs M., Zantomasi A., Szabo A. (2019). Porcine Hepatic Response to Fumonisin B1 in a Short Exposure Period: Fatty Acid Profile and Clinical Investigations. Toxins.

[27] Yang S., Li L., Yu L., Sun L., Li K., Tong C., Xu W., Cui G., Long M., Li P. (2020). Selenium-enriched yeast reduces caecal pathological injuries and intervenes changes of the diversity of caecal microbiota caused by Ochratoxin-A in broilers. Food Chem. Toxicol..

[28] Zhou Y., Qi S., Meng X., Lin X., Duan N., Zhang Y., Yuan W., Wu S., Wang Z. (2021). Deoxynivalenol photocatalytic detoxification products alleviate intestinal barrier damage and gut flora disorder in BLAB/c mice. Food Chem. Toxicol..

[29] Wang J., He Y., Yu D., Jin L., Gong X., Zhang B. (2020). Perilla oil regulates intestinal microbiota and alleviates insulin resistance through the PI3K/AKT signaling pathway in type-2 diabetic KKAy mice. Food Chem. Toxicol..

[30] Hillman M., Weström B., Aalaei K., Erlanson-Albertsson C., Wolinski J., Lozinska L., Sjöholm I., Rayner M., Landin-Olsson M. (2019). Skim milk powder with high content of Maillard reaction products affect weight gain, organ development and intestinal inflammation in early life in rats. Food Chem. Toxicol..

[31] Hills R.D., Pontefract B.A., Mishcon H.R., Black C.A., Sutton S.C., Theberge C.R. (2019). Gut Microbiome: Profound Implications for Diet and Disease. Nutrients.

[32] Claesson M.J., Jeffery I.B., Conde S., Power S.E., O’Connor E.M., Cusack S., Harris H.M., Coakley M., Lakshminarayanan B., O’Sullivan O. (2012). Gut microbiota composition correlates with diet and health in the elderly. Nature.

[33] Antonissen G., Van Immerseel F., Pasmans F., Ducatelle R., Janssens G.P., De Baere S., Mountzouris K.C., Su S., Wong E.A., De Meulenaer B. (2015). Mycotoxins Deoxynivalenol and Fumonisins Alter the Extrinsic Component of Intestinal Barrier in Broiler Chickens. J. Agric. Food Chem..

[34] Zhang F., Chen Z., Jiang L., Chen Z., Sun H. (2021). Response of Fecal Bacterial Flora to the Exposure of Fumonisin B1 in BALB/c Mice. Toxins.

[35] Gibiino G., Lopetuso L.R., Scaldaferri F., Rizzatti G., Binda C., Gasbarrini A. (2018). Exploring Bacteroidetes: Metabolic key points and immunological tricks of our gut commensals. Dig. Liver Dis..

[36] Zhang Y., Liu Y., Li J., Xing T., Jiang Y., Zhang L., Gao F. (2020). Dietary corn-resistant starch suppresses broiler abdominal fat deposition associated with the reduced cecal Firmicutes. Poult. Sci..

[37] Chen Y.-H., Chiu C.-C., Hung S.-W., Huang W.-C., Lee Y.-P., Liu J.-Y., Huang Y.-T., Chen T.-H., Chuang H.-L. (2019). Gnotobiotic mice inoculated with Firmicutes, but not Bacteroidetes, deteriorate nonalcoholic fatty liver disease severity by modulating hepatic lipid metabolism. Nutr. Res..

[38] Yang F., Zhu W.-J., Edirisuriya P., Ai Q., Nie K., Ji X.-M., Li Y., Zhou K. (2022). Beneficial effects of a combination of Clostridium cochlearium and Lactobacillus acidophilus on body weight gain, insulin sensitivity, and gut microbiota in high-fat diet–induced obese mice. Nutrition.

[39] Demirci M., Bahar Tokman H., Taner Z., Keskin F.E., Çağatay P., Ozturk Bakar Y., Özyazar M., Kiraz N., Kocazeybek B.S. (2020). Bacteroidetes and Firmicutes levels in gut microbiota and effects of hosts TLR2/TLR4 gene expression levels in adult type 1 diabetes patients in Istanbul, Turkey. J. Diabetes Complicat..

[40] Li F., Wang J., Huang L., Chen H., Wang C. (2017). Effects of Adding *Clostridium* sp. WJ06 on Intestinal Morphology and Microbial Diversity of Growing Pigs Fed with Natural Deoxynivalenol Contaminated Wheat. Toxins.

[41] Daniel S., Pusadkar V., McDonald J., Mirpuri J., Azad R.K., Goven A., Lund A.K. (2021). Traffic generated emissions alter the lung microbiota by promoting the expansion of Proteobacteria in C57Bl/6 mice placed on a high-fat diet. Ecotoxicol. Environ. Saf..

[42] Vasques-Monteiro I.M.L., Silva-Veiga F.M., Miranda C.S., de Andrade Gonçalves É.C.B., Daleprane J.B., Souza-Mello V. (2021). A rise in Proteobacteria is an indicator of gut-liver axis-mediated nonalcoholic fatty liver disease in high-fructose-fed adult mice. Nutr. Res..

[43] Zhang Y., Zhang P., Shang X., Lu Y., Li Y. (2021). Exposure of lead on intestinal structural integrity and the diversity of gut microbiota of common carp. Comp. Biochem. Physiol. Part C Toxicol. Pharmacol..

[44] Martínez Pizarro S. (2021). Transplantation of fecal microbiota in multidrug-resistant Klebsiella pneumoniae colonization and infection. Gastroenterología y Hepatología.

[45] Workneh M., Wang F., Romagnoli M., Simner P.J., Carroll K. (2016). Bypass graft infection and bacteremia caused by *Anaerostipes* caccae: First report of human infection caused by a recently described gut anaerobe. Anaerobe.

[46] Schelstraete W., Devreese M., Croubels S. (2020). Comparative toxicokinetics of Fusarium mycotoxins in pigs and humans. Food Chem. Toxicol..

[47] Chang J., Wang T., Wang P., Yin Q., Liu C., Zhu Q., Lu F., Gao T. (2020). Compound probiotics alleviating aflatoxin B1 and zearalenone toxic effects on broiler production performance and gut microbiota. Ecotoxicol. Environ. Saf..

[48] Bolger A.M., Lohse M., Usadel B. (2014). Trimmomatic: A flexible trimmer for Illumina sequence data. Bioinformatics.

[49] Reyon D., Tsai S.Q., Khayter C., Foden J.A., Sander J.D., Joung J.K. (2012). FLASH assembly of TALENs for high-throughput genome editing. Nat. Biotechnol..

[50] Caporaso J.G., Kuczynski J., Stombaugh J., Bittinger K., Bushman F.D., Costello E.K., Fierer N., Pena A.G., Goodrich J.K., Gordon J.I. (2010). QIIME allows analysis of high-throughput community sequencing data. Nat. Methods.

[51] Wang Q., Garrity G.M., Tiedje J.M., Cole J.R. (2007). Naive Bayesian classifier for rapid assignment of rRNA sequences into the new bacterial taxonomy. Appl. Environ. Microbiol..

