# Supplementary material for: Effects of Fumonisin B and Hydrolyzed Fumonisin B on Growth and Intestinal Microbiota in Broilers
Source: Toxins (Basel). 2022 Feb 23;14(3):163. doi: 10.3390/toxins14030163 (PMC8954478; doi:10.3390/toxins14030163)
Supplement: Supplementary file 1 [file toxins-14-00163-s001.zip › toxins-1513712-supplementary.pdf]

toxins

MDPI

*Article*

# Effects of Fumonisin B and Hydrolyzed Fumonisin B on Growth and Intestinal Microbiota in Broilers

**Song Yu , Bingxuan Jia, Huikang Lin, Shuo Zhang, Dianzhen Yu, Na Liu and Aibo Wu**

**Table S1.** FBs and HFBs affect the body weight of broilers.

| | ANOVA with Tukey's multiple comparisons test | Significant | P-Value |
|---|---|---|---|
| Week 6 | Control vs. L-FBs | ns | 0.9315 |
| | Control vs. H-FBs | ns | 0.1385 |
| | Control vs. L-HFBs | ns | 0.9991 |
| | Control vs. H-HFBs | ns | 0.1507 |
| | L-FBs vs. H-FBs | * | 0.0174 |
| | L-FBs vs. L-HFBs | ns | 0.9783 |
| | L-FBs vs. H-HFBs | * | 0.0196 |
| | H-FBs vs. L-HFBs | ns | 0.0600 |
| | H-FBs vs. H-HFBs | ns | >0.9999 |
| | L-HFBs vs. H-HFBs | ns | 0.0666 |
| Week 7 | Control vs. L-FBs | ns | 0.7984 |
| | Control vs. H-FBs | *** | 0.0002 |
| | Control vs. L-HFBs | ns | 0.8260 |
| | Control vs. H-HFBs | *** | 0.0006 |
| | L-FBs vs. H-FBs | * | 0.0129 |
| | L-FBs vs. L-HFBs | ns | >0.9999 |
| | L-FBs vs. H-HFBs | * | 0.0278 |
| | H-FBs vs. L-HFBs | ** | 0.0059 |
| | H-FBs vs. H-HFBs | ns | 0.9992 |
| | L-HFBs vs. H-HFBs | * | 0.0140 |
| Week 8 | Control vs. L-FBs | ns | 0.8592 |
| | Control vs. H-FBs | **** | <0.0001 |
| | Control vs. L-HFBs | ns | 0.9819 |
| | Control vs. H-HFBs | **** | <0.0001 |
| | L-FBs vs. H-FBs | **** | <0.0001 |
| | L-FBs vs. L-HFBs | ns | 0.9887 |
| | L-FBs vs. H-HFBs | *** | 0.0009 |
| | H-FBs vs. L-HFBs | **** | <0.0001 |
| | H-FBs vs. H-HFBs | ns | 0.9241 |
| | L-HFBs vs. H-HFBs | **** | <0.0001 |

**Table S2.** Differentially expressed bacteria after FBs and HFBs treatment.

| ANOVA | Kruskal Wallis |
|---|---|
| Phylum | |
| | Elusimicrobia |
| | Planctomycetes |
| Class | |
| Elusimicrobia | Elusimicrobia |
| Phycisphaerae | Phycisphaerae |
| | Subgroup_22 |
| Order | |
| WD260 | WD260 |
| Deltaproteobacteria_Incertae_Sedis | Deltaproteobacteria_Incertae_Sedis |
| Subgroup_13 | Subgroup_13 |
| Reyranellales | Reyranellales |
| Gastranaerophilales | Gastranaerophilales |
| Subgroup_2 | Subgroup_2 |
| Acetobacterales | Acetobacterales |
| Actinomarinales | JG36-TzT-191 |
| SJA-28 | SAR324_clade(Marine_group_B) |
| MVP-88 | Rickettsiales |
| Mollicutes_RF39 | R7C24 |
| uncultured_Acidobacteriales_bacterium | Enterobacteriales |
| | KF-JG30-C25 |
| | uncultured_soil_bacterium |
| | Izimaplasmatales |
| | Rhodospirillales |
| | Lactobacillales |
| Family | |
| Candidatus_Nitrosoarchaeum_limnia_SFB1 | Candidatus_Nitrosoarchaeum_limnia_SFB1 |
| SM2D12 | SM2D12 |
| uncultured_Bradyrhizobiaceae_bacterium | uncultured_Bradyrhizobiaceae_bacterium |
| Prolixibacteraceae | Prolixibacteraceae |
| uncultured_forest_soil_bacterium | uncultured_forest_soil_bacterium |
| Mycobacteriaceae | Mycobacteriaceae |
| Geodermatophilaceae | Geodermatophilaceae |
| Magnetospirillaceae | Magnetospirillaceae |
| Rhizobiales_Incertae_Sedis | Rhizobiales_Incertae_Sedis |
| Reyranellaceae | Reyranellaceae |
| Tannerellaceae | Clostridiales_vadinBB60_group |
| S15-21 | uncultured_Acidothermaceae_bacterium |
| Unknown_Family | Acetobacteraceae |
| Chitinimonadaceae | Arcobacteraceae |
| Ilumatobacteraceae | Paenibacillaceae |
| | Saccharospirillaceae |
| | Rs-E47_termite_group |
| | metagenome |
| | Nitrosococcaceae |
| | SAR116_clade |
| | Enterobacteriaceae |
| | Inquilinaceae |
| | Acidothermaceae |
| | A21b |
| | URHD0088 |
| | Phaseolus_acutifolius_(tepary_bean) |
| | Lactobacillaceae |
| Genus | |
| GOUTA6 | GOUTA6 |
| Magnetospirillum | Magnetospirillum |
| uncultured_Rickettsiales_bacterium | uncultured_Rickettsiales_bacterium |

| | |
|---|---|
| Candidatus_Nitrosoarchaeum_limnia_SFB1 | Candidatus_Nitrosoarchaeum_limnia_SFB1 |
| Sunxiuqinia | Sunxiuqinia |
| uncultured_Bradyrhizobiaceae_bacterium | uncultured_Bradyrhizobiaceae_bacterium |
| Lachnospiraceae_UCG-010 | Lachnospiraceae_UCG-010 |
| Erysipelotrichaceae_UCG-003 | Erysipelotrichaceae_UCG-003 |
| Klebsiella | Klebsiella |
| uncultured_forest_soil_bacterium | uncultured_forest_soil_bacterium |
| Clostridium_sensu_stricto_1 | Clostridium_sensu_stricto_1 |
| Lachnospiraceae_UCG-006 | Lachnospiraceae_UCG-006 |
| Anaerostipes | Anaerostipes |
| Deferrisoma | Deferrisoma |
| Ornithinimicrobium | Ornithinimicrobium |
| Chitiniphilus | Chitiniphilus |
| Erysipelothrix | Erysipelothrix |
| Mycobacterium | Mycobacterium |
| Undibacterium | Undibacterium |
| Granulicella | Granulicella |
| uncultured_Nitrosomonadales_bacterium | uncultured_Nitrosomonadales_bacterium |
| Flavonifractor | Paenibacillus |
| PAUC26f | wb1-P19 |
| Draconibacterium | Segetibacter |
| Azovibrio | Lachnospiraceae_NK4B4_group |
| Chitinimonas | WCHB1-32 |
| Pseudolabrys | Lechevalieria |
| Amycolatopsis | Lachnospiraceae_AC2044_group |
| Conexibacter | Polaromonas |
| [Eubacterium]_ventriosum_group | Duganella |
| Azohydromonas | Roseiarcus |
| Ruminococcaceae_UCG-010 | Azospirillum |
| Parabacteroides | uncultured_proteobacterium |
| Ruminococcaceae_UCG-003 | Kibdelosporangium |
| | Plesiomonas |
| | BIyi10 |
| | Roseburia |
| | Mycoplasma |
| | GAS113 |
| | Rheinheimera |
| | Edaphobacter |
| | [Eubacterium]_brachy_group |
| | Glutamicibacter |
| | Roseomonas |
| | Blastococcus |
| | Inquilinus |
| | Acidothermus |
| | Eggerthella |
| | Lactobacillus |
| | Phaseolus_acutifolius_(tepary_bean) |
| | Anaerofilum |
| | Sandaracinus |
| | uncultured_Rhizomicrobium_sp. |
| | Xanthobacter |
| | Fournierella |
| | Lachnospiraceae_NK4A136_group |
| | Oleibacter |
| | Dyella |
| | Triticum_aestivum_(bread_wheat) |
| | Arcobacter |
| | Acidipila |
| | Turneriella |
| | uncultured_Acidothermaceae_bacterium |

| | | |
|---|---|---|
| | | uncultured_Nitrosomonadaceae_bacterium |
| | | uncultured_Hyphomicrobiaceae_bacterium |
| | | Clostridium_sensu_stricto_8 |
| Species | | |
| | uncultured_Rickettsiales_bacterium | uncultured_Rickettsiales_bacterium |
| | Candidatus_Nitrosoarchaeum_limnia_SFB1 | Candidatus_Nitrosoarchaeum_limnia_SFB1 |
| | uncultured_forest_soil_bacterium | uncultured_forest_soil_bacterium |
| | Bacteroides_acidifaciens_JCM_10556 | Bacteroides_acidifaciens_JCM_10556 |
| | Lactobacillus_salivarius | Lactobacillus_salivarius |
| | uncultured_rumen_bacterium | uncultured_rumen_bacterium |
| | uncultured_Nitrosomonadales_bacterium | uncultured_Nitrosomonadales_bacterium |
| | uncultured_spirochete | uncultured_Dongia_sp. |
| | uncultured_organism | uncultured_Acidothermaceae_bacterium |
| | uncultured_soil_bacterium | uncultured_Nitrosomonadaceae_bacterium |
| | | uncultured_Burkholderia_sp. |
| | | Rhizobium_leguminosarum |
| | | uncultured_Desulfovibrio_sp. |
| | | uncultured_Polaromonas_sp. |
| | | uncultured_Gemmatimonadaceae_bacterium |
| | | Lactobacillus_gasseri |
| | | uncultured_Hyphomicrobiaceae_bacterium |
| | | Phaseolus_acutifolius_(tepary_bean) |
| | | uncultured_Rhizomicrobium_sp. |
| | | uncultured_Bradyrhizobiaceae_bacterium |
| | | Other |
| | | uncultured_Lactobacillaceae_bacterium |
| | | uncultured_Allobaculum_sp. |
| | | Lactobacillus_vaginalis |
| | | Triticum_aestivum_(bread_wheat) |

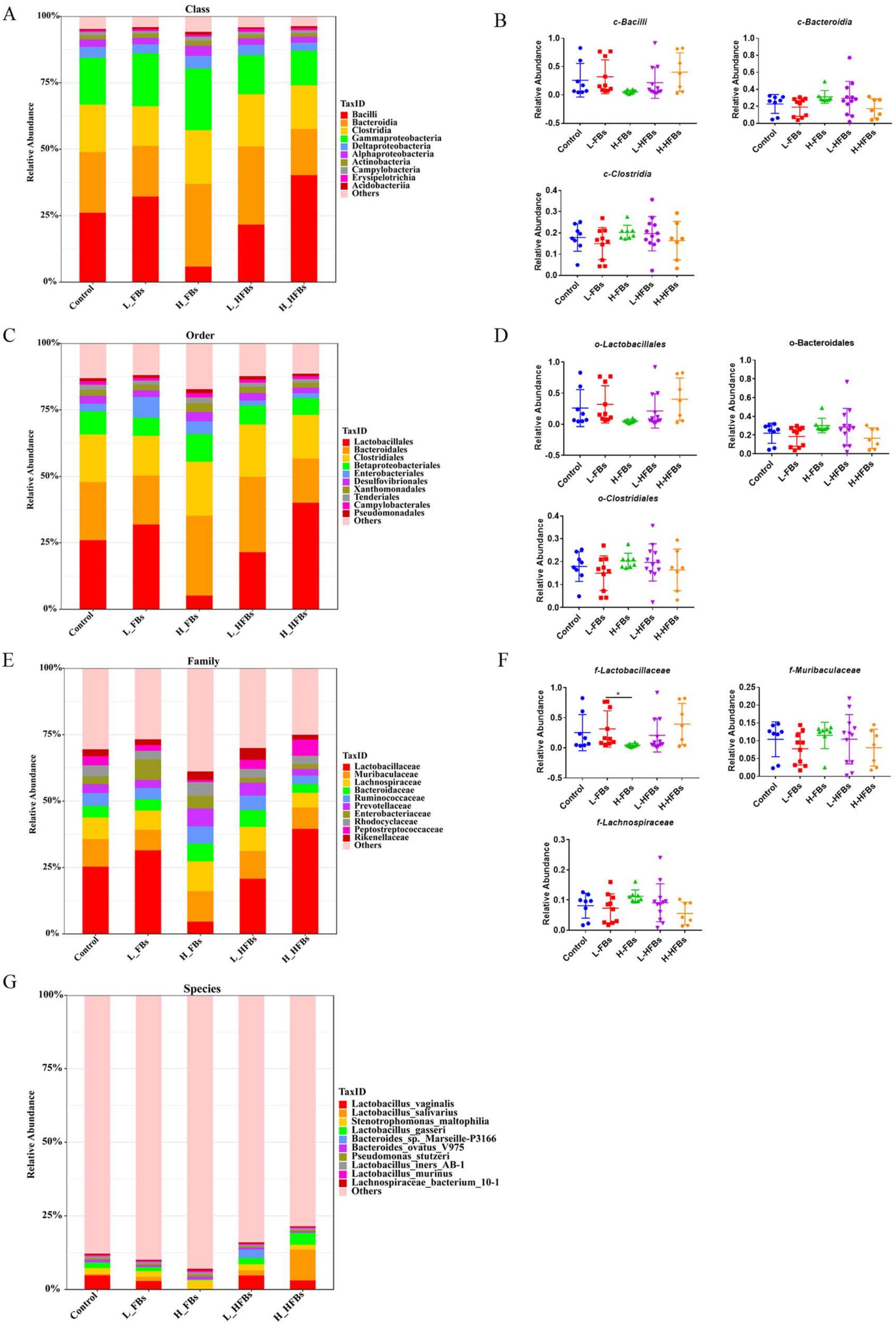


**Figure S1.** Relative abundance of bacteria and analysis of dominant bacteria at Class (**A-B**), Order (**C-D**), Family (**E-F**) and Species (**G**) level.

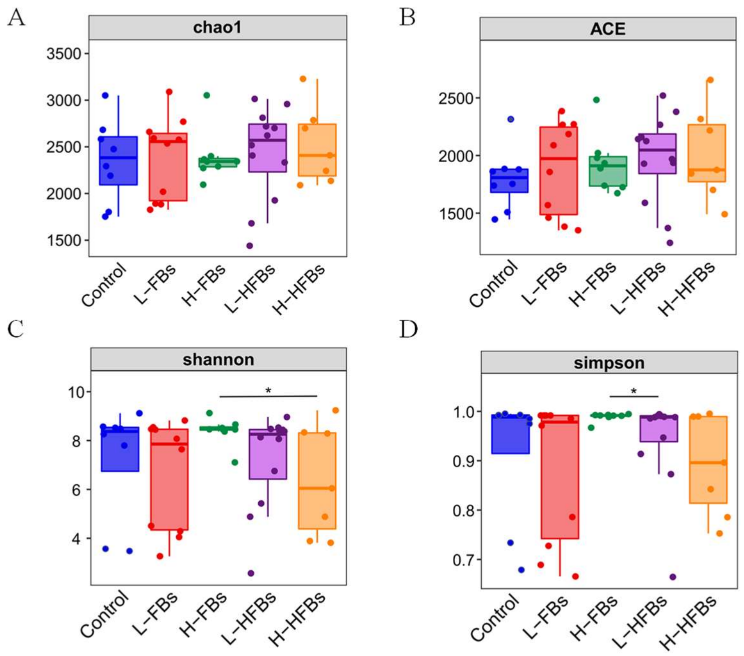


**Figure S2.** Alpha analysis of intestinal microbiota after FBs and HFBs treatment. (**A**) chao 1 analysis. (**B**) ACE analysis. (**C**) shannon analysis. (**D**) simpson analysis.
